# Supplementary material for: A Genome-Scale Metabolic Model for Methylococcus capsulatus (Bath) Suggests Reduced Efficiency Electron Transfer to the Particulate Methane Monooxygenase
Source: Front Microbiol. 2018 Dec 4;9:2947. doi: 10.3389/fmicb.2018.02947 (PMC6288188; doi:10.3389/fmicb.2018.02947)

Genome-scale Metabolic Map

High-resolution image of the genome-scale metabolic map of iMcBath. This metabolic map was constructed using escher (*escher.github.io*) and is available at *github.com/ChristianLieven/memote-m-capsulatus*

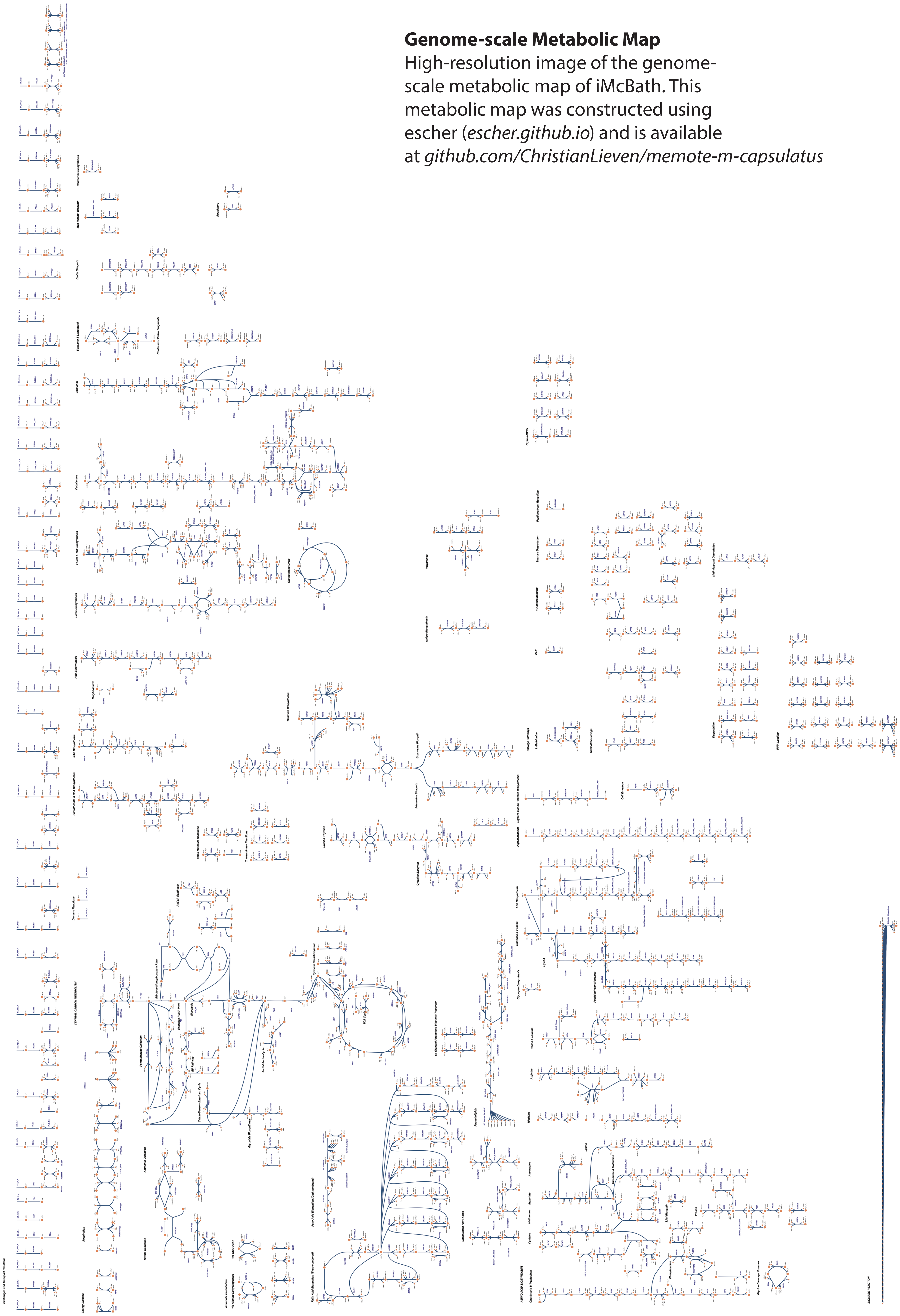

Supplement: FIGURE S1 — Genome-scale Metabolic Map. High-resolution image of the genome-scale metabolic map of iMcBath. The metabolic map was constructed using escher. [file Image_1.pdf]
