# Supplementary material for: A Genome-Scale Metabolic Model for Methylococcus capsulatus (Bath) Suggests Reduced Efficiency Electron Transfer to the Particulate Methane Monooxygenase
Source: Front Microbiol. 2018 Dec 4;9:2947. doi: 10.3389/fmicb.2018.02947 (PMC6288188; doi:10.3389/fmicb.2018.02947)
Supplement: TABLE S1 — Stoichiometry of the biomass reaction in iMcBath. Table showing the stoichiometry and references of all components in the biomass reaction. [file Table_1.docx]

The metabolic model iCL730 and scripts that were used to construct it, in addition to the scripts that were used for the analysis presented in this work, are provided at https://github.com/ChristianLieven/memote-m-capsulatus.

Supplemental Table 1: Stoichiometry of the biomass reaction in iCL730.

|  | Compound | | mmol gDCW^-1^ | References |
| --- | --- | --- | --- | --- |
| Proteins |  | |  |  |
|  | Ala | | 0.576 | unibio.dk^9^, Øverland et al, 2010^10^ |
|  | Arg | | 0.254 | ‘’ |
|  | Asp | | 0.468 | ‘’ |
|  | Cys | | 0.038 | ‘’ |
|  | Glu | | 0.519 | ‘’ |
|  | Gly | | 0.484 | ‘’ |
|  | His | | 0.104 | ‘’ |
|  | Ile | | 0.242 | ‘’ |
|  | Leu | | 0.415 | ‘’ |
|  | Lys | | 0.277 | ‘’ |
|  | Met | | 0.130 | ‘’ |
|  | Phe | | 0.185 | ‘’ |
|  | Pro | | 0.248 | ‘’ |
|  | Ser | | 0.246 | ‘’ |
|  | Thr | | 0.271 | ‘’ |
|  | Trp | | 0.093 | ‘’ |
|  | Tyr | | 0.132 | ‘’ |
|  | Val | | 0.360 | ‘’ |
|  | Gln | | 0.150 | de la Torre et al, 2015^4^ |
|  | Asn | | 0.119 | ‘’ |
| Nucleic Acids | *Ribonucleic acid (RNA)* | |  |  |
|  | A | | 0.024 | unibio.dk; Ward et al,2004^11^ |
|  | U | | 0.026 | ‘’ |
|  | G | | 0.041 | ‘’ |
|  | C | | 0.044 | ‘’ |
|  | *Deoxyribonucleic acid (DNA)* | |  |  |
|  | A | | 0.009 | ‘’ |
|  | T | | 0.009 | ‘’ |
|  | G | | 0.014 | ‘’ |
|  | C | | 0.016 | ‘’ |
| Lipids | *Phospholipids* | |  |  |
|  | PE | | 0.081 | Average of 4 experiments by Makula 1978, converted from measurements in µmol/g DW^12^ |
|  | PG | | 0.014 | ‘’ |
|  | PC | | 0.008 | ‘’ |
|  | CL | | 0.003 | ‘’ |
|  | Sterols | |  |  |
|  | Squalene | | 0.013 | Bird et al. ,1971^13^ |
|  | Diplopterol | | 0.013 | Estimated |
|  | Lanosterol | | 0.005 | Bird et al. ,1971; Zymosterol was detected |
|  | Fatty acids | |  |  |
|  | C14:0 Myristic acid | | 0.001 | unibio.dk, Müller et al, 2004^14^ |
|  | C14:1 | | 0.000 | ‘’ |
|  | C15:0 | | 0.000 | ‘’ |
|  | C16:0 Palmitic acid | | 0.016 | ‘’ |
|  | C17:00 | | 0.002 | ‘’ |
|  | Cyc17:0 | | 0.002 | ‘’ |
|  | C18:0 Stearic acid | | 0.000 | ‘’ |
|  | C18:1 Oleic acid | | 0.000 | ‘’ |
| Salts |  | |  |  |
|  | Phosphorous | | 0.200 | unibio.dk |
|  | Sulphur | | 0.056 | ‘’ |
|  | Chloride | | 0.214 | ‘’ |
|  | Calcium | | 0.070 | ‘’ |
|  | Potassium | | 0.176 | ‘’ |
|  | Magnesium | | 0.123 | ‘’ |
|  | Sodium | | 0.039 | ‘’ |
|  | Iron | | 0.006 | ‘’ |
|  | Copper | | 0.002 | ‘’ |
|  | Zinc | | 0.001 | ‘’ |
|  | Cobalt | | 0.000 | ‘’ |
|  | Nickel | | 0.000 | ‘’ |
|  | Manganese | | 0.000 | ‘’ |
| Cell Wall |  | |  |  |
|  | Peptidoglycan | | 0.053 | ‘’ |
|  | LPS | | 0.002 | ‘’ |
|  | Glucose | | 0.250 | ‘’ |
| Vitamins |  | |  |  |
|  | Thiamin B1 | | 0.000 | ‘’ |
|  | Riboflavin B2 | | 0.000 | ‘’ |
|  | Nicotinic acid | | 0.001 | ‘’ |
|  | Inositol 30 | | 0.000 | ‘’ |
| Intracellular Metabolites | |  | |  |
|  | Ribulose 5-phospate | | 0.001 | de la Torre et al, 2015 |
|  | Fructose-1,6-bisphosphate | | 0.001 | ‘’ |
|  | Fructose-6-phospate | | 0.003 | ‘’ |
|  | Glucose-6-phosphate | | 0.002 | ‘’ |
|  | Glyceraldehyde-3-phosphate/ dhap | | 0.003 | ‘’ |
|  | 6-phospogluconic acid | | 0.000 | ‘’ |
|  | 2-dehydro-3-deoxy-phosphogluconate | | 0.000 | ‘’ |
|  | Phosphoglycerate | | 0.006 | ‘’ |
|  | Phosphoenolpyruvate | | 0.005 | ‘’ |
|  | Pyruvate | | 0.015 | ‘’ |
|  | Acetyl-CoA | | 0.000 | ‘’ |
|  | Succinate | | 0.002 | ‘’ |
|  | Malate | | 0.004 | ‘’ |
|  | Fumarate | | 0.001 | ‘’ |
|  | Citrate | | 0.001 | ‘’ |
|  | Glycerate | | 0.001 | ‘’ |
|  | Nitrate | | 0.002 | unibio.dk |
|  | Nitrite | | 0.002 | ‘’ |
| Energy Requirement | |  | |  |
|  | ADP | | -23.087 | Calculated from Thiele and Palsson, 2010 |
|  | Pi | | -23.087 | ‘’ |
|  | Ppi | | -0.183 | ‘’ |
|  | H | | -23.087 | ‘’ |
|  | H2O | | 17.776 | ‘’ |
|  | ATP | | 23.087 | ‘’ |
